# High charge of cerebroid nests in nodular melanomas predicts tumor aggressiveness and high mutational tumoral burden: a pilot study

**DOI:** 10.3389/fonc.2024.1336895

**Published:** 2024-07-19

**Authors:** Stefania Caramaschi, Alessandro Mangogna, Laura Bertoni, Marco Manfredini, Francesca Farnetani, Paola Parente, Vito Attino, Gerardo Cazzato, Tiziana Salviato, Giovanni Pellacani, Luca Reggiani Bonetti

**Affiliations:** ^1^ Clinical and Experimental Medicine PhD Program, Department of Biomedical, Metabolic, and Neural Sciences, University of Modena and Reggio Emilia, Modena, Italy; ^2^ Department of Medical and Surgical Sciences for Children and Adults, University of Modena and Reggio Emilia, Modena, Italy; ^3^ Institute of Pathologic Anatomy, Department of Medicine, University of Udine, Udine, Italy; ^4^ Department of Surgery, Medicine, Dentistry and Morphological Sciences with Interest in Transplant, Oncology and Regenerative Medicine, University of Modena and Reggio Emilia, Modena, Italy; ^5^ Dermatology Unit, Department of Surgery, Medicine, Dentistry and Morphological Sciences with Interest in Transplant, Oncology and Regenerative Medicine, University of Modena and Reggio Emilia, Modena, Italy; ^6^ Unit of Pathology, Fondazione Istituto di Ricovero e Cura a Carattere Scientifico (IRCCS) Ospedale Casa Sollievo della Sofferenza, San Giovanni Rotondo, Foggia, Italy; ^7^ Section of Molecular Pathology, Department of Emergency and Organ Transplantation (DETO), University of Bari “Aldo Moro”, Bari, Italy; ^8^ Pathology Unit, University Hospital of Modena, Modena, Italy; ^9^ Dermatology Clinic, Department of Clinical Internal, Anesthesiological and Cardiovascular Sciences, Sapienza University of Rome, Roma, Italy

**Keywords:** nodular melanoma, cerebroid nests, melanocytes, *BRAF*, prognosis

## Abstract

**Purpose:**

Even today, melanoma is a highly aggressive neoplasm with a high mortality rate. The nodular type is very aggressive and has cerebroid nests of melanocytes (CNMs) at the growth edge, morphologically similar to the poorly differentiated neoplastic epithelial cell clusters described in colorectal, breast, and endometrioid endometrial cancers.

**Patients and methods:**

We selected 25 nodular melanomas (NMs) with known molecular profiles, of which the entire paraffin-embedded lesion was available. We counted CNMs under a microscopic at a magnification of 20x (i.e., a microscopic field with a major axis of 1 mm). Based on the number of CNMs in the area, melanomas were classified into three groups: G1 (CNMs ranging from 0 to 4), G2 (CNMs ranging from 5 to 9), and G3 (CNMs ≥ 10). The presence of CNMs and their counts were compared with molecular and histopathological data.

**Results:**

Seventeen (NMs) were grouped as G1 (68%), 5 as G2 (20%), and 3 as G3 (12%) based on CNMs count. The presence of CNMs correlated with epithelioid cell morphology (*p* < 0.05), Clark IV and V levels (*p* < 0.05), vascular invasion (*p* < 0.05), and biological mutants (*p* < 0.05). Melanomas with ≥ 10 CNMs more frequently show ulceration (*p* < 0.02) and the BRAF V600E mutation (*p* < 0.02).

**Conclusion:**

CNMs count has a predictive role regardless of tumor size; their association with the BRAF V600E mutation suggests their predictive significance in response to biologics. However, further investigations are needed to strengthen this hypothesis.

## Introduction

Malignant melanomas are the most aggressive skin cancers, with increasing morbidity in recent years. They include heterogeneous neoplasms characterized by different dermatoscopic, histological, and molecular profiles (1–3). A particular type of melanoma is the nodular variant, characterized by deep growth, frequent metastasis, and a high rate of genetic aberrations (4–6). Histologically, nodular melanomas (NMs) are composed of large, atypical spindle or epithelioid melanocytes, pleomorphic, sometimes organized in aggregates morphologically attributable to cerebroid nests of melanocytes (CNMs). They are mainly described in the peripheral portion of the tumors, where they assume an infiltrative profile or within the tumor mass. CNMs are round or oval in shape and are composed of aggregates of at least 5 undifferentiated atypical melanocytes, primarily devoid of pigmentation (7, 8). However, no attention has been paid to the count of nests detected in the neoplasm. No specific correlation has been investigated between their maximum concentration and clinical-pathological features.

In nodular melanoma, CNMs could be compared to poorly differentiated clusters (PDCs) of tumor cells. PDCs have been identified at the growing edge of the tumor in some types of epithelial cancers, including colorectal, gastric, breast, and endometrioid endometrial cancers (9–12). By definition, they are composed of ≥ 5 undifferentiated cells and are counted in the microscopic field under a × 20 objective lens (i.e., a microscopic field of 1 mm) (9, 13). In colorectal cancer, their highest number observed identifies the grade of malignancy: < 5 clusters for grade 1 (G1), 5 to 9 clusters for grade 2 (G2), and 10 or more clusters for grade 3 (G3) (14). The high number of PDCs is strongly associated with lymphatic vascular invasion and lymph node metastases, irrespective of the TNM stage (14–16). The correlation between the number of PDCs and the depth of infiltration of the submucosa in colorectal and gastric tumors is relevant (10, 14, 17). The unfavorable prognostic significance in terms of survival, demonstrated in studies with large case series of colorectal and gastric cancers, encourages to consider PDCs as possible tools in assessing the risk of lymph node involvement and progressive disease, regardless of pTNM stages and other histological features (10, 18). *KRAS*, *NRAS* and *BRAF* mutant colorectal carcinomas tend to form a high number of PDCs suggesting that the state of genomic instability may be a condition favoring their formation (19).

The aim of this preliminary study is to investigate the CNMs in a series of 25 NMs and to describe their association with their biological and histological characteristics.

## Materials and methods

### Case selection and histological analysis

Twenty-five NMs were selected from the archive of the Unit of Pathology of Modena University from 2000 to 2020. Clinical information, including sex, age at diagnosis, tumor location and size, and biological profile, were collected. Three expert pathologists independently reviewed hematoxylin and eosin (H&E) stained slides representative of the melanomas (LRB, LB & PP). The following data were collected: tumor cell morphology (epithelioid or spindle), Breslow thickness, Clark’s level, mitosis x mm^2^, ulceration, lymphovascular invasion, and tumor-infiltrating lymphocytes (TILs).

### Cerebroid nests of melanocytes

CNMs morphology and count were defined according to the definition of PDC proposed by Ueno et al. in colorectal cancer (13). Thus, we described CNMs as round or oval aggregates of undifferentiated epithelioid atypical melanocytes, primarily devoid of pigmentation, composed of at least 5 tumor cells, detected in hematoxylin and eosin (H&E) stained slides. We evaluated the entire surface of the tumors and its peripheral zone, identifying the highest CNMs concentration – hot spot under a microscopic field of an objective lens 20x (i.e., a microscopic field with a major axis of 1 mm). First CNMs evaluation was performed in a dichotomy system (present-absent) defining CNMs+ melanomas and CNMs- melanomas. Thus, according to a significant number of CNMs at 20x, we grouped melanomas into grade G1-CNMs+ melanoma (0 – 4 CNMs), grade G2-CNMs+ melanoma (5 – 9 CNMs), and grade G3-CNMs melanoma (≥ 10 CNMs). We distinguished central CNMs (cCNMs) from peripheral CNMs (pCNMs) concerning the primary neoplastic lesion.

### Statistical analysis

Statistical analysis was performed with STATA software, version 14 (Stata Corp LP 4905 Lakeway Drive College Station, Texas 77845 USA). Qualitative data were expressed as frequency and percentage. The Chi-square test (Fisher’s exact test) examined the relationship among qualitative variables. A *p*-value < 0.05 was considered significant.

## Results

The study included 25 NMs: 13 patients were males and 12 females, with a mean age of 67 years old (range 34 – 75 years). In detail, the mean diameter of the melanomas was 1.8 cm (range 0.9 – 2.3 cm), and tumor location included limbs (12 cases; 48%), chest-abdomen (8 cases; 32%), and other skin areas (5 cases; 20%). Breslow thickness was ≤ 1.0 mm in 13 cases (52%) (all pT1b) and > 1.0 mm in 12 (48%); Clark’s levels III, IV, and V were reached by 8 (32%), 15 (60%), and 2 (8%) tumors, respectively.

Epithelioid morphology of the tumor cells was observed in 16 cases (64%), and spindle cells in 9 of them (36%); 22 tumors (88%) showed > 5 mitosis x mm^2^; 6 masses (24%) were ulcerated. Lymph vascular invasion was observed in 12 cases (48%); TILs were present in 14 tumors (56%).

Eleven melanomas (44%) showed mutations. *BRAF* (V600E) mut was present in 6 cases (24%), *NRAS* (Q61R) mut in 4 cases (16%), and *c-KIT* (EXE11-G565E) mut in 1 case (4%).

**Figure 1:**
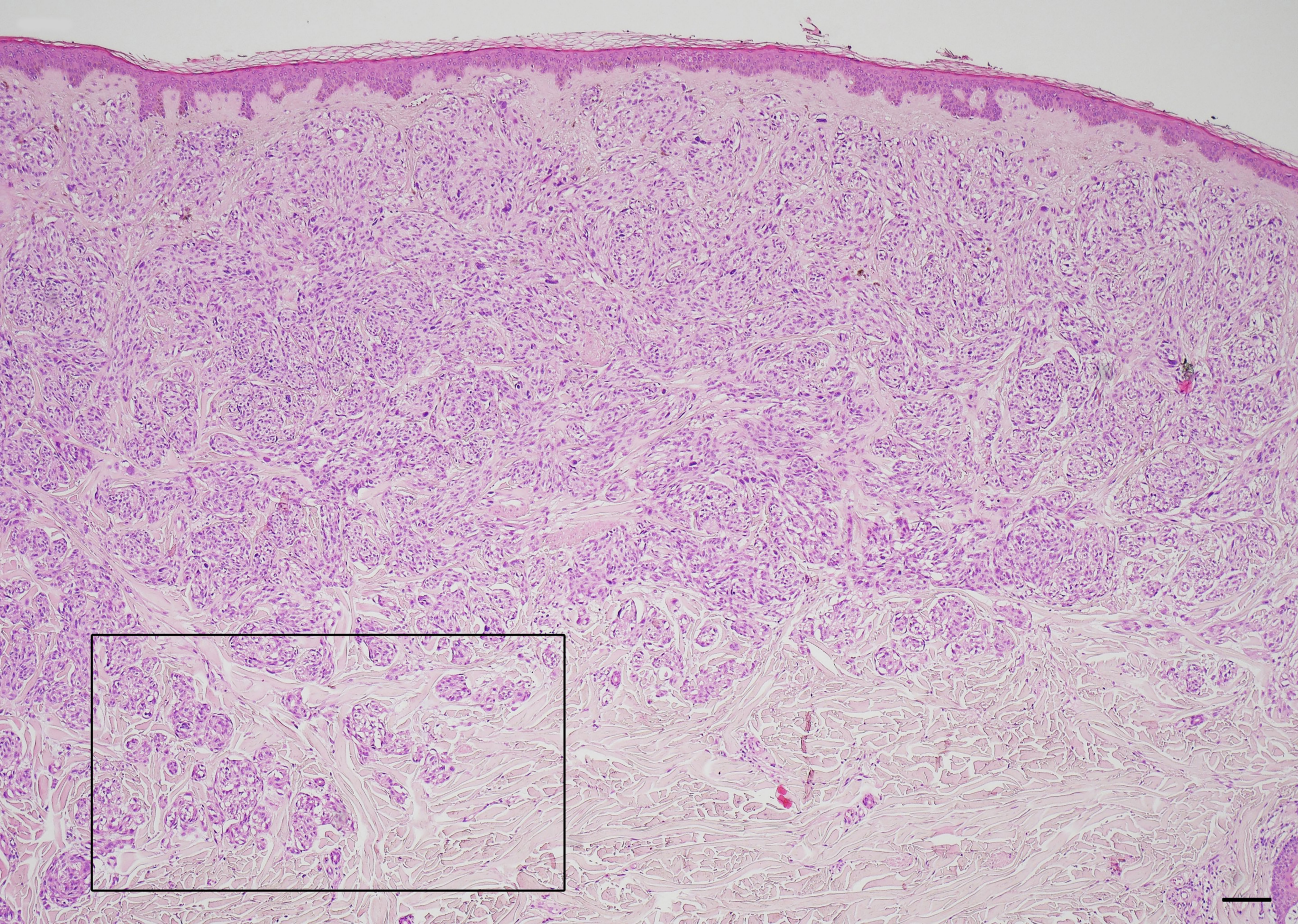
Cerebroid nests of melanocytes at the periphery – growth edge – of nodular melanoma. Hematoxylin and eosin stained slide. Magnification 4x, scale bars 200 µm.

**Figure 2:**
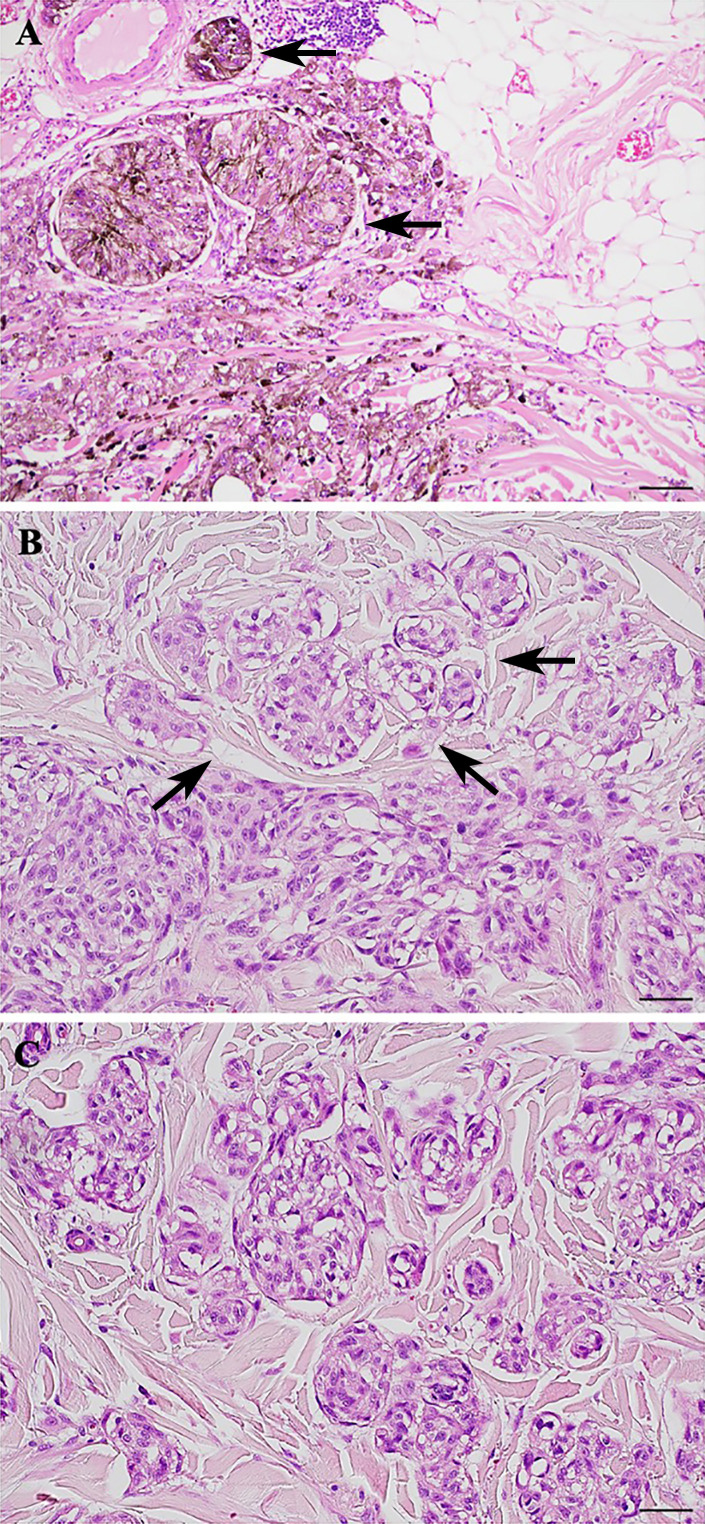
Cerebroid nests of melanocytes (CNMs) grading in nodular melanoma. **(A)** CNMs G1 (from 0 to 4 buds). **(B)** CNMs G2 (from 5 to 9 buds). **(C)** CNMs G3 (≥ 10 buds). Hematoxylin and eosin stained slide. Magnification 20x, scale bars 100 µm.

CNMs were observed in the periphery of 15 melanomas (60%) (**Figure 1**). Six of them (40%) were also detected within the tumor mass. The higher number of CNMs at 20x was seen mainly at the peripheral zone of the melanoma and, according to this, 17 melanomas (68%) were classified as G1 (0 – 4 CNMs), 5 (20%) as G2 (5 – 9 CNMs), and 3 (12%) as G3 (10 or more CNMs) (**Figures 2A–C**).

Histological and molecular features of the CNM+ and CNM- melanomas are listed in **Table 1**. In detail, CNM+ tumors were mainly epithelioid in morphology (*p* = 0.041), showed more depth invasion (*p* = 0.042), showed more frequently lymph vascular invasion (*p* = 0.022) and a mutated status (*p* = 0.048); CNM+ melanomas were larger in diameter, although no statistical significance was reached (*p* = 0.068).

**Table 1:** Correlation between cerebroid nests of melanocytes and clinico-pathological features.

	CNM+	CNM-	*p*-value	*X^2^* value
Tumor dimension				
≤ 1 cm	1	1		
1.1 – 2 cm	4	7		
> 2 cm	10	2	0.068352	5.3662
Cell morphology				
Epithelioid	12	4		
Spindle	3	6	0.041227	4.1667
Breslow thickness				
≤ 1.0 mm	6	7		
> 1.0 mm	9	3	0.244934	0.2491
Clark levels				
Level III	3	5		
Level IV	3	4		
Level V	9	1	0.04296	6.2946
Ulceration of the surface				
Presence	5	1		
Absence	10	9	0.18084	1.7909
Mitoses				
Presence	14	8		
Absence	1	2	0.3148	1.0101
Mutational status				
Mutated tumors	9	2		
Wild type tumors	6	8	0.0484	3.8961
*BRAF* gene				
Mutated (V600E)	5	1		
Wild type	10	9	0.1808	1.7909
*NRAS* gene				
Mutated (Q61R)	3	1		
Wild type	12	9	0.5041	0.4464
*c-KIT* gene				
Mutated (EXE11-G565E)	1	0		
Wild type	0	0	n.a.	n.a.
Lymphovascular invasion				
Detected	10	2		
Non detected	5	8	0.0221	5.235
Tumor infiltrating lymphocytes (TILs)				
Brisk pattern	10	4		
Non-brisk pattern	5	6	0.1882	1.7316

Chi-square test (X^2^); significance p-value < 0.05.

CNMs, Cerebroid nests of melanocytes.

n.a., not available.

Histological and molecular features are listed in **Table 2**, G1-CNM+, G2-CNM+, and G3-CNM+ melanomas, respectively. High-grade CNM+ melanomas (G3-CNM+) were more frequently ulcerated (*p* = 0.025), and *BRAF* (V600E) mutated (*p* = 0.028). Although statistical significance was not reached, they exhibited predominantly epithelioid cell morphology, and were lymph vascular invasive.

**Table 2:** Correlation between cerebroid nests of melanocytes grading and clinico-pathological features.

	G1 (0 – 4 CNMs)	G2 (5 – 9 CNMs)	G3(≥ 10 CNMs)	*p*-value	*X^2^* value
Tumor dimension					
≤ 1 cm	1	0	1		
1.1 – 2 cm	7	2	2		
> 2 cm	9	3	0	0.7402	1.9757
Cell morphology					
Epithelioid	15	3	3		
Spindle	2	2	0	0.3162	2.3039
Breslow thickness					
≤ 1.0 mm	8	3	2		
> 1.0 mm	9	2	1	0.5991	1.0244
Clark levels					
Level III	6	2	0		
Level IV	11	3	1		
Level V	0	0	2	0.1811	6.252
Ulceration of the surface					
Presence	2	1	3		
Absence	15	4	0	0.0256	7.3272
Mitoses					
Presence	15	4	3		
Absence	2	1	0	0.7634	8.673
Mutational status					
Mutated tumors	6	2	3		
Wild type tumors	11	3	0	0.3498	2.1005
*BRAF* gene					
Mutated (V600E)	2	1	3		
Wild type	15	4	0	0.0287	7.1014
*NRAS* gene					
Mutated (Q61R)	4	0	0		
Wild type	0	0	0	n.a	n.a
*c-KIT* gene					
Mutated (EXE11-G565E)	0	1	0		
Wild type	0	0	0	n.a.	n.a.
Lymphovascular invasion					
Detected	7	2	3		
non detected	10	3	0	0.4527	1.5849
Tumor infiltrating lymphocytes (TILs)					
Brisk pattern	8	4	2		
Non-brisk pattern	9	1	1	0.3947	1.8589

Chi-square test (X^2^); significance p-value < 0.05.

CNMs, Cerebroid nests of melanocytes.

n.a., not available.

## Discussion

**Figure 3:**
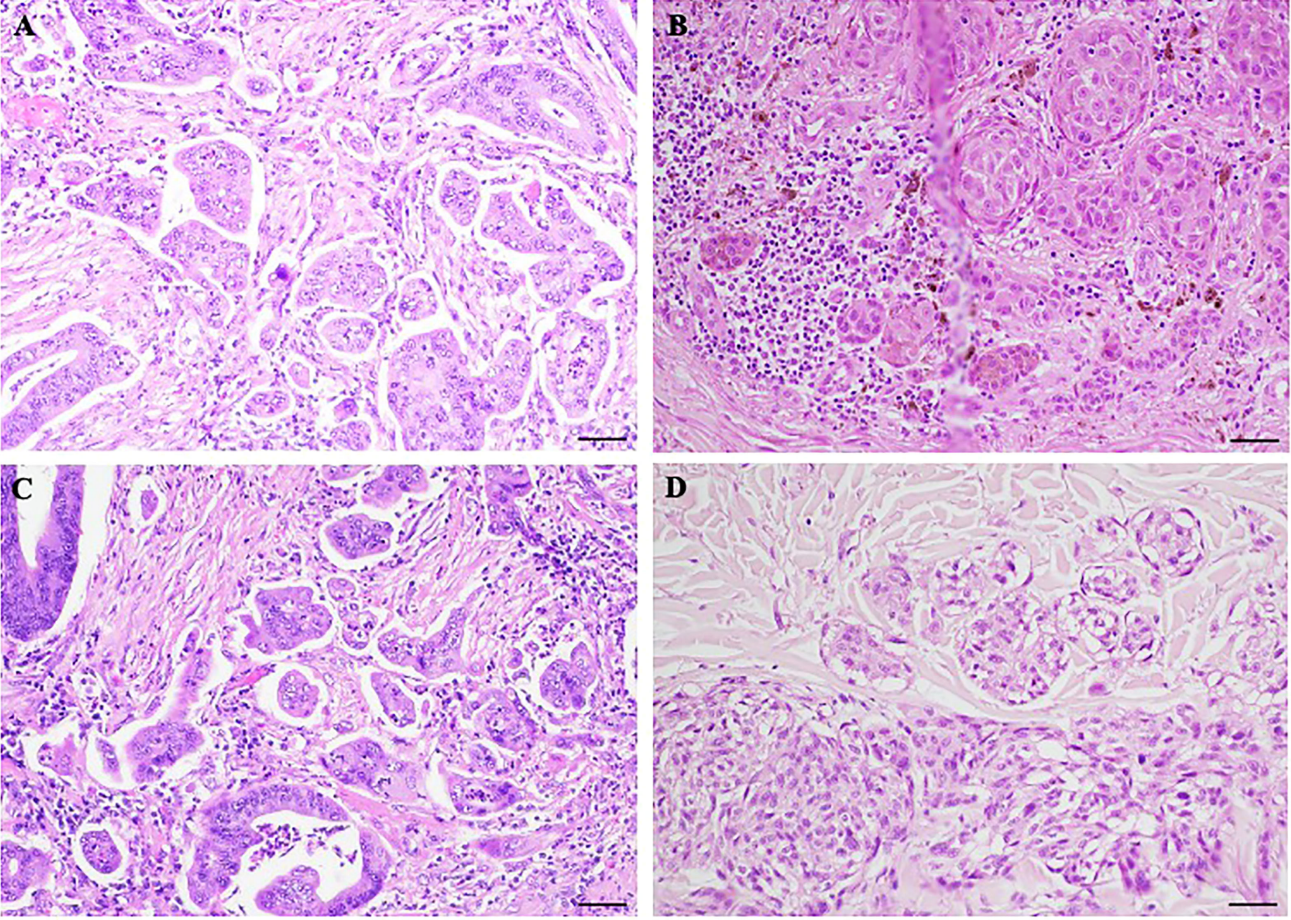
Morphological comparison between poorly differentiated clusters (PDCs) and cerebroid nests of melanocytes (CNMs). **(A–C)** PDCs in colorectal cancer. **(B–D)** CNMs in nodular melanoma. Hematoxylin and eosin stained slide. Magnification 20x, scale bars 100 µm.

NM represents a clinically aggressive histologic variant characterized by atypical pleomorphic melanocytic cells with large cytoplasm, spindle or epithelioid shape, and prominent nucleoli (3, 20, 21). In many tumors in the periphery, CNMs can be detected, which detach from the main mass, taking on an infiltrative appearance. CNMs are readily detectable in slides stained with H&E and show a morphology similar to the aggregates of PDCs observed in colorectal carcinoma (9). Although CNMs are described in NMs, no studies currently consider their histopathologic or prognostic significance. Concerning this, we examined the histological slides of a selected group of NMs and demonstrated the presence of CNMs at the periphery of 15 (60%) of them and a number ≥ 10 nests at 20x field in 3 cases (12%). We showed that CNMs were significantly associated with lymphatic vascular invasion, and higher Clark level, suggesting a possible role in tumor growth. This would be responsible for vascular invasion and, thus, metastasis, typical of their more aggressive clinical behavior (20, 22–28). Therefore, CNMs represent the active growth front of these melanomas, typically observed in epithelial malignancies. In support of this, it is interesting to report that CNMs were significantly associated with the epithelioid phenotype of the melanoma from which they originated. As shown in **Figure 3**, where we compare PDCs and CNMs, we hypothesize that NMs with epithelioid morphology have an aggressive growth attitude, probably comparable to poorly differentiated epithelial neoplasms with active neoplasm “buds” (17). Indeed, in our experience reported here, CNM+ melanomas reach Clark’s IV and V levels of infiltration and are clinically characterized by recurrence and metastasis (*p* = 0.011; **Table 2**). This aspect of invasiveness has also been similarly demonstrated in colorectal cancers with a high number of PDCs in tumors deeply infiltrating the intestinal wall (pT3 and pT4 tumors) (15, 16, 29).

CNMs are more abundant at the periphery of melanomas than those detectable within masses, which are rarer to identify. As surmised for colorectal PDCs (30, 31), this could indicate that their origin derives from the epithelio-stroma interaction, which belongs to the mechanisms that drive cell growth and proliferation (30). This aspect is typically observed in epithelial tumors (32) and therefore now acceptable as a possible explanation to the formation of PDCs (14). It must, however, be transferred with caution in the context of NMs with CNMs since the origin is neuroectodermal and for that reason melanoma cells may not be subject to epithelial-mesenchymal transition (EMT)-mediated effects (33). On the other hand, it is also known that in the dermal invasive phase, tumor melanocytes acquire molecular changes in cell-cell adhesion proteins, including the downregulation of the junctional protein E-cadherin (33–35) and that this process becomes particularly evident during the transition from radial to vertical growth phase (36). On the other hand, it should be mentioned that nonepithelial tumors, including melanoma, can acquire mesenchymal-like properties (37). However, it is still not entirely clear how transcription factors that induce EMT drive the growth and progression of nonepithelial tumors, such as melanoma (37, 38).

In recent years, it has been identified that genetic factors, including activating mutations in the *BRAF* and *NRAS* oncogenes, contribute to melanoma initiation, promoting its growth and metastasis, as well as the transition of melanoma cells into different epithelial and mesenchymal states (39). Our study reported that *BRAF* (V600E) mutated NMs have a higher number of CNMs at the periphery of the mass, suggesting that the accumulation of genetic mutations in tumor cells could promote the creation of clones of cells that can aggregate and migrate by invading the dermis. Studies on PDCs and colorectal cancer report that a high number of PDCs in colon cancer are associated with the V600E mutation in the *BRAF* gene, although without statistical significance due to the limited number of cases collected (14, 19). BRAF is a Ras-activated serine/threonine protein kinase that participates in the MAP kinase/ERK signaling pathway (40), mutated in approximately 50% of melanomas [Catalogue of Somatic Mutations in Cancer (COSMIC) at http://www.sanger.ac.uk/cosmic] (41). *BRAF* (V600E) mut has been implicated in melanoma progression, senescence evasion, apoptosis, uncontrolled replication potential, and angiogenesis, resulting in tissue invasion and metastasis (42). The interaction between melanocytic tumor cells and the surrounding microenvironment, consisting of different extracellular matrix components and growth factors, and the induction of EMT in melanoma are influenced by common mutations and/or deregulated expression of BRAF, NRAS, and PTEN, which appear to act synergistically with each other and with different microenvironmental factors (43). The microenvironment can integrate aberrant genetic changes such as mutations in *BRAF* to promote melanomagenesis and support an invasive cell phenotype. Therefore, *BRAF* mutation could promote the genesis of tumor melanocyte clones that would take on the biomolecular characteristics required to generate CNMs (40, 44).

Our study has some limitations: first, we have a “small” series of NMs, and second, molecular mutations were detected in only 11 out of 25 cases, but this is due to the difficulty in enrolling cases with CNMs as well as the fact that this is a pilot study, so the results should be considered preliminary.

In conclusion, CNMs represent a promising additional unfavorable histologic feature of NMs with epithelioid morphology. Because CNMs+ melanomas appear to be associated with the *BRAF* V600E mutation, the presence of CNMs could have cancer predictive significance for identifying a subgroup of patients who might benefit from specific biologic drugs. However, further studies are needed to confirm this hypothesis.

## Data availability statement

The original contributions presented in the study are included in the article/supplementary material. Further inquiries can be directed to the corresponding author.

## Ethics statement

The study was approved by the Ethical Review Committee of the Modena University Hospital (protocol number CE 289\13) and conducted according to the Helsinki Declaration. Written informed consent was obtained from each patient enrolled. The studies were conducted in accordance with the local legislation and institutional requirements. Written informed consent for participation in this study was provided by the participants’ legal guardians/next of kin.

## Author contributions

SC: Writing – original draft. AM: Writing – original draft, Writing – review & editing. LB: Conceptualization, Formal analysis, Writing – original draft, Writing – review & editing. MM: Visualization, Writing – review & editing. FF: Visualization, Writing – review & editing. PP: Visualization, Writing – review & editing. VA: Visualization, Writing – review & editing. GC: Visualization, Writing – review & editing. TS: Writing – review & editing. GP: Visualization, Writing – review & editing. LRB: Conceptualization, Formal analysis, Writing – original draft, Writing – review & editing.
